# Atezolizumab Plus Carboplatin and Etoposide in Patients with Untreated Extensive-Stage Small-Cell Lung Cancer: Interim Results of the MAURIS Phase IIIb Trial

**DOI:** 10.1093/oncolo/oyad342

**Published:** 2024-02-20

**Authors:** Emilio Bria, Floriana Morgillo, Marina Chiara Garassino, Fortunato Ciardiello, Andrea Ardizzoni, Alessio Stefani, Francesco Verderame, Alessandro Morabito, Antonio Chella, Giuseppe Tonini, Marina Gilli, Ester Del Signore, Rossana Berardi, Manlio Mencoboni, Alessandra Bearz, Angelo Delmonte, Marta Rita Migliorino, Cesare Gridelli, Antonio Pazzola, Manuela Iero, Filippo De Marinis

**Affiliations:** Comprehensive Cancer Center, Fondazione Policlinico Universitario Agostino Gemelli IRCCS, Università Cattolica del Sacro Cuore, Rome, Italy; Medical Oncology and Haematology, Department of Precision Medicine, University of Campania “Luigi Vanvitelli,” Naples, Italy; Department of Hematology/Oncology, The University of Chicago, Chicago, IL, USA; Medical Oncology, Department of Precision Medicine, University of Campania Luigi Vanvitelli, Naples, Italy; Division of Medical Oncology, IRCCS Azienda Ospedaliero-Universitaria di Bologna, Bologna, Italy; Medical Oncology, Università Cattolica del Sacro Cuore, Rome, Italy; Medical Oncology Unit, Ospedale Cervello Villa Sofia, Palermo, Italy; Thoracic Medical Oncology, National Tumor Institute “Fondazione G Pascale,” IRCCS, Naples, Italy; Pneumology Unit, Azienda Ospedaliero Universitaria Pisana, Pisa, Italy; Department of Oncology, Campus Bio-Medico University, Rome, Italy; Unit of Pulmonary Oncology, University of Campania “L. Vanvitelli,” Monaldi Hospital, Naples, Italy; Division of Thoracic Oncology, European Institute of Oncology, IRCCS, Milan, Italy; Department of Medical Oncology, Università Politecnica delle Marche, Ancona, Italy; Oncology Unit, Villa Scassi Hospital, ASL3-Genovese, Genoa, Italy; Department of Medical Oncology, Centro di Riferimento Oncologico di Aviano (CRO), IRCCS, Aviano, Italy; IRCCS Istituto Romagnolo per lo Studio dei Tumori (IRST) “Dino Amadori,” Meldola, Italy; Pulmonary Oncology Unit, Azienda Ospedaliera San Camillo Forlanini Hospital, Rome, Italy; Division of Medical Oncology, A.O.S.G. Moscati, Avellino, Italy; Medical Oncology Unit, University Hospital (AOU) of Sassari, Sassari, Italy; Medical Dep-Oncology, Roche S.p.A., Monza, Italy; Thoracic Oncology Division, European Institute of Oncology (IEO), IRCCS, Milan, Italy

**Keywords:** atezolizumab, chemotherapy, induction, extensive-stage small cell lung cancer

## Abstract

**Background:**

MAURIS is an Italian multicenter, open-label, phase IIIb ongoing trial, aiming at evaluating the safety and effectiveness of atezolizumab + carboplatin/etoposide in patients with newly diagnosed, extensive-stage small-cell lung cancer (ES-SCLC). The primary objective is the safety evaluation.

**Materials and Methods:**

Patients received atezolizumab + carboplatin/etoposide Q3W for 4-6 cycles in the induction phase, followed by atezolizumab maintenance Q3W. We presented the interim analysis on safety (referring to the induction phase) and clinical effectiveness, in all patients (*N* = 154) and in subgroups that received ≤3 (*N* = 23), 4 (*N* = 43), and 5-6 cycles (*N* = 89) of induction.

**Results:**

At a median follow-up of 10.5 months, 139 patients (90.3%) discontinued treatment. Serious adverse events occurred in 29.9% of patients overall, and the rate was lower in patients with 5-6 cycles (19.1%) than in those with 4 (34.9%) or ≤3 (63.6%) cycles. Immune-mediated adverse events were reported in 14.9%, 15.7%, 11.6%, and 18.2% of patients, overall and by subgroup, respectively. The median overall survival and progression-free survival were 10.7 and 5.5 months, respectively. Overall, 111 patients (71.6%) had a tumor response.

**Conclusions:**

Interim results provide further evidences about safety and efficacy profile of atezolizumab + carboplatin/etoposide treatment in a ES-SCLC patient population closer to that observed in clinical practice.

**Clinical Trial Registration:**

Eudract No. 2019-001146-17, NCT04028050.

## Teaser

This analysis is based on interim data from the MAURIS study, an Italian multicenter, phase IIIb ongoing trial to evaluate treatment with atezolizumab plus carboplatin/etoposide in patients with newly diagnosed extensive-stage small-cell lung cancer.

**Implications for Practice**

This analysis, based on interim data from the MAURIS study, an Italian multicenter, phase IIIb ongoing trial, aiming at evaluating treatment with atezolizumab + carboplatin/etoposide in patients with newly diagnosed extensive-stage small-cell lung cancer (ES-SCLC) provides preliminary evidence that safety and efficacy results from the regulatory trial (ie, IMpower133) may be replicable in a patient population of ES-SCLC with more relaxed selection criteria, closer to that of “real life” clinical practice.

## Introduction

Small-cell lung cancer (SCLC) accounts for approximately 15% of all lung cancers and is characterized by early development of metastatic disease and hence poor prognosis.^1^ Although limited-stage SCLC can be treated with chemotherapy and radiation with the potential for long-term survival,^2^ the majority (approximately 70%) of patients with SCLC are diagnosed with extensive-stage disease (ES-SCLC), which has poor survival prospects (median overall survival [OS] approximately 10 months).^3^

Up to recent years, the standard first-line treatment for patients with ES-SCLC has been platinum-based chemotherapy with etoposide, a topoisomerase II inhibitor.^4^ The advanced knowledge of the biology of SCLC has led to the development of new therapeutic options, including targeted therapies and immunotherapies. Atezolizumab is a humanized IgG1 monoclonal antibody that targets programmed death-ligand 1 (PD-L1) and inhibits the interaction between PD-L1 and its receptors, PD-1 and B7-1 (also known as CD80), both of which function as inhibitory receptors expressed on T cells. Therapeutic blockade of PD-L1 binding by atezolizumab has been shown to enhance the magnitude and quality of tumor-specific T-cell responses, resulting in improved antitumor activity.^5,6^

Targeting the PD-L1 pathway with atezolizumab has demonstrated activity in patients with advanced malignancies who have failed standard-of-care therapies. Atezolizumab has been and is currently studied as a single-agent in the advanced cancer and adjuvant/neoadjuvant therapy settings, as well as in combination with chemotherapy, targeted therapy, and cancer immunotherapy. The drug is now approved in the European Union for the treatment of urothelial carcinoma, non–small cell lung cancer, SCLC, triple-negative breast cancer, and hepatocellular carcinoma.^7^

Atezolizumab has been the first immune checkpoint inhibitor to be approved, in combination with carboplatin and etoposide, for the treatment of adult patients with ES-SCLC.^8^ Approval was based on primary data from the multinational phase I/III IMpower133 trial in PD-L1-unselected patients with previously untreated ES-SCLC, ECOG 0-1, and treated asymptomatic brain metastases,^9^ which showed that the addition of atezolizumab to 4 cycles of carboplatin and etoposide in ES-SCLC was associated with significantly longer OS and progression-free survival (PFS) compared to chemotherapy alone, with a safety profile consistent with the known safety profile of the individual agents.^10^ Study updates have shown that the benefit of atezolizumab versus placebo and the safety profile was maintained at a median follow-up for OS of approximately 2 years,^11^ in the subset of patients who reached the maintenance phase of the study^12^ and in the elderly population as compared to their younger counterpart.^13^

Recently, several trials conducted in single countries that have evaluated the effects of atezolizumab plus chemotherapy in patients with ES-SCLC treated in the real-world setting^14-20^ have shown that the results of the IMpower133 trial^9^ are reproducible in patients with potentially worse prognosis treated in standard clinical practice.

MAURIS is a multicenter, open-label, single-arm, phase IIIb trial conducted in Italy aimed at evaluating the safety and efficacy of atezolizumab + carboplatin-etoposide in patients with newly diagnosed ES-SCLC aimed at assessing safety and efficacy in a patient population closer to “real world” clinical setting as compared to the registrative trial. This article describes the findings of the interim data analysis conducted after 1 year from the end of enrolment.

## Patients and Methods

### Patients

The study population included patients with histologically or cytologically confirmed ES-SCLC and measurable disease, life expectancy >12 weeks, ECOG PS 0-2, no previous systemic treatment for ES-SCLC, and adequate hematologic and end organ function. Patients with asymptomatic treated or untreated brain metastasis could be eligible. The full list of inclusion and exclusion criteria is presented in the Supplementary Material.

### Treatments

Patients received atezolizumab 1200 mg + carboplatin (AUC 5 mg/ml/minute) and etoposide (100 mg/m^2^ BSA on days 1-3 of each cycle) every 3 weeks for 4-6 cycles (according to investigator choice) in the induction phase, followed by atezolizumab maintenance every 3 weeks up to disease progression, unacceptable toxicity or clinical deterioration. Following disease progression per RECIST v1.1, treatment could be continued in case of evidence of clinical benefit, absence of symptoms, and signs (including laboratory values, such as new or worsening hypercalcemia) indicating unequivocal progression of the disease, absence in decline in ECOG PS due to disease progression, and absence of tumor progression at critical anatomical sites (eg, leptomeningeal disease). Prophylactic or therapeutic anticoagulation therapy, corticosteroids administered for chronic obstructive pulmonary disease or asthma, low-dose corticosteroids administered for orthostatic hypotension or adrenocortical insufficiency, palliative radiotherapy not interfering with the assessment of tumor target lesions and local therapy (eg, surgery, stereotactic radiosurgery, radiotherapy, radiofrequency ablation) were permitted during the study. Premedication with antihistamines, antipyretics, and/or analgesics could be administered from the second atezolizumab infusion. Use of the following treatments was not permitted: other anticancer therapy, live attenuated vaccines, systemic immunostimulant, or immunosuppressive therapy.

### Outcome Measures

The primary endpoints of the study were: incidence of serious adverse events (SAEs) and of immune-mediated adverse events (imAEs, list in Supplementary Material), defined as those AEs consistent with an immune-mediated mechanism of action requiring treatment with systemic corticosteroids or hormone replacement therapy. Secondary endpoints were incidence of adverse events of special interest (AESI, list in Supplementary Material), AEs associated with drug-induced liver injury (DILI, list in Supplementary Material), survival rate at 1 year, overall survival (OS, time from start of treatment to death from any cause), progression-free survival (PFS, time from start of treatment to the first occurrence of disease progression or death from any cause, whichever occurs first), and overall response rate (ORR, including complete response or patient response according to RECIST v1.1). Tumor assessments were conducted at screening, every 6 weeks for the first 48 weeks following cycle 1, and every 9 weeks thereafter until the occurrence of disease progression. Patients who continued treatment beyond disease progression continued to undergo tumor assessments every 6 weeks until study treatment was discontinued. Treatment-emergent adverse events (TEAEs) were assessed according to the National Cancer Institute Common Terminology Criteria for Adverse Events, version 5.0.

### Statistics

Safety analyses were conducted in the safety set, defined as all enrolled patients who had at least one administration of atezolizumab + carboplatin/ etoposide. The results of safety refer to the induction phase of the study, overall safety data will be reported in the final analysis. Efficacy analyses were conducted in the intent-to-treat (ITT) set, defined as all enrolled patients. Data analysis was performed in overall patients and in subgroups based on the number of cycles of induction, ie, in patients receiving ≤3 cycles, 4 cycles, and 5-6 cycles. There was no formal statistical hypothesis, hence all safety (primary) endpoints results are presented by 95% CIs and descriptively explained. However, a sample size of 150 patients produces a 2-sided 95% CI with a width equal to 0.165 when the sample AEs incidence is equal to 50% (maximum variability). Time-dependent variables (OS and PFS) were analyzed using Kaplan-Meier methods: medians with 95% CI were calculated, with the number and percentage of the patients with event or censored. AE terms were assigned to a preferred term (PT) and were classified by the primary system organ class (SOC) according to the Medical Dictionary for Regulatory Activities (MedDRA) thesaurus, version 23.0.

### Ethics

The study protocol was approved by the reference Ethic Committee of each investigational study site before the study started. Patients gave their written informed consent to study participation before the start of any study-related procedure.

## Results

### Patient Disposition and Baseline Characteristics

From August 2019 to July 2020, 155 patients were enrolled (ITT set) in 25 sites in Italy: 154 (99.4%) patients started treatment (safety set) and 119 (76.8%) entered the maintenance phase. Overall, 139 patients (90.3% of safety set) discontinued treatment, 35 patients (22.7%) in the induction phase, and 104 (67.5%) in the maintenance phase (Supplementary Fig. S1). Reasons for discontinuation of treatments and discontinuation of study are presented in Supplementary Table S1.

At the cutoff date for the interim analysis (clinical data cutoff September 6, 2021, data snapshot taken on October 28, 2021), the median duration of follow-up was 10.5 months (range 0-2-24.3 months). In the safety set, 89 patients (57.8%) received 5-6 cycles, 22 (14.3%) received ≤3 cycles, and 43 (27.9%) received 4 cycles of induction. The median number of cycles was 8.0 (range 1-35) for atezolizumab and 6.0 (range 1-6) for carboplatin and etoposide. Fourteen patients received thoracic radiotherapy during the study and 15 received prophylactic cranial irradiation during the maintenance phase. Overall, 76 patients (49.0%) received immunostimulants during the study: 54 patients (34.8%) received filgrastim, 18 (11.6%) pegfilgrastim, 3 (1.9%) granulocyte-colony stimulating factors, and 2 (1.3%) lipegfilgrastim.

Table 1 shows the baseline characteristics of patients overall and by subgroups based on length of induction. Patients who received 5-6 cycles of induction (*N* = 89) were younger, included fewer males, more patients with ECOG PS of 0, fewer patients with hepatic metastases and previous radiotherapy, than those that received ≤3 (*N* = 23) or 4 (*N* = 43) cycles of induction.

**Table 1.** Summary of baseline characteristics of patients overall and by subgroup (ITT set)

	Total *n* = 155	≤3 cycles *n* = 23	4 cycles *n* = 43	5-6 cycles *n* = 89
Age (years), mean (SD)	65.1 (9.05)	67.2 (7.48)	67.2 (9.90)	63.6 (8.77)
Gender, *n* (%)				
Females	60 (38.7%)	5 (21.7%)	15 (34.9%)	40 (44.9%)
Males	95 (61.3%)	18 (78.3%)	28 (65.1%)	49 (55.1%)
ECOG PS, *n* (%)				
0	68 (44.2%)	5 (21.7%)	19 (44.2%)	44 (50.0%)
1	80 (51.9%)	16 (69.6%)	23 (53.5%)	41 (46.6%)
2	6 (3.9%)	2 (8.7%)	1 (2.3%)	3 (3.4%)
Missing	0 (0.0%	0 (0.0%)	0 (0.0%)	1 (1.1%)
Smoking status, *n* (%)				
Never smoked	8 (5.2%)	1 (4.3%)	2 (4.7%)	5 (5.6%)
Current smoker	59 (38.1%)	9 (39.1%)	17 (39.5%)	33 (37.1%)
Former smoker	88 (56.8%)	13 (56.5%)	24 (55.8%)	51 (57.3%)
Previous diagnosis of limited stage SCLC, *n* (%)	14 (9.0%)	2 (8.7%)	8 (18.6%)	4 (4.5%)
Brain metastases at baseline, *n* (%)	19 (12.3%)	5 (21.7%)	3 (7.0%)	11 (12.4%)
Untreated brain metastases at baseline, *n* (%)	13 (8.4%)	2 (8.7%)	2 (4.7%)	9 (10.1%)
Liver metastases at baseline, *n* (%)	86 (55.5%)	14 (60.9%)	29 (67.4%)	43 (48.3%)
Presence of comorbidities, *n* (%)				
Coronary artery disease	4 (4.5%)	1 (4.3%)	1 (2.3%)	2 (4.7%)
Diabetes	15 (9.7%)	3 (13.0%)	3 (7.0%)	9 (10.1%)
Hypertension	85 (54.8%)	14 (60.9%)	25 (58.1%)	46 (51.7%)
Prior chemotherapy/non-anthracycline, *n* (%)	11 (7.1%)	1 (4.3%)	5 (11.6%)	5 (5.6%)
Prior radiotherapy, *n* (%)	19 (12.3%)	6 (26.1%)	6 (14.0%)	7 (7.9%)
Prior cancer-related surgery, *n* (%)	11 (7.1%)	1 (4.3%)	7 (16.3%)	3 (3.4%)
Longest diameter of target lesions (mm), mean (SD)	49.0 (32.1)	50.2 (35.8)	47.4 (30.0)	49.5 (32.2)

### Safety

Table 2 shows the overall summary of results of safety (induction phase) overall and by subgroup in the induction phase. The results of primary endpoints showed that SAEs were reported in 29.9% of patients overall and were related to treatment in 18.2% of patients. Incidence of SAEs in the induction phase was lower in patients who received 5-6 cycles than in those who received 4 or ≤3 cycles. Serious TEAEs by SOC, PT, and maximum grade are presented in Supplementary Table S2. Neutropenia (17 patients, 9.8%) was the most common serious TEAE by PT in the overall safety population. None of the other serious TEAEs by PT was reported in more than 2% of patients. Only 3 serious TEAEs were considered to be related only to atezolizumab (one case of diarrhea, one of encephalitis, and one of pruritus).

**Table 2.** Overall summary of results of safety in the induction phase (safety set)

	Total *n* = 154	≤3 cycles *n* = 22	4 cycles *n* = 43	5-6 cycles *n* = 89
TEAEs	139 (90.3%)	21 (95.5%)	41 (95.3%)	77 (86.5%)
Treatment-related TEAEs	117 (76.0%)	13 (59.1%)	35 (81.4%)	69 (77.5%)
Grades 3-4 TEAEs	80 (51.9%)	13 (59.1%)	23 (53.5%)	44 (49.4%)
Grades 3-4 treatment-related TEAEs	73 (47.4%)	10 (45.5%)	22 (51.2%)	41 (46.1%)
SAEs	46 (29.9%)	14 (63.6%)	15 (34.9%)	17 (19.1%)
Treatment-related SAEs	28 (18.2%)	6 (27.3%)	12 (27.9%)	10 (11.2%)
Treatment discontinuations due to TEAEs	9 (5.8%)	5 (22.7%)	1 (2.3%)	3 (3.4%)
Atezolizumab discontinuations due to TEAEs	7 (4.5%)	5 (22.7%)	1 (2.3%)	1 (1.1%)
Carboplatin discontinuations due to TEAEs	7 (4.5%)	4 (18.2%)	0 (0.0%)	3 (3.4%)
Etoposide discontinuations due to TEAEs	7 (4.5%)	4 (18.2%)	0 (0.0%)	3 (3.4%)
TEAEs leading to death	7 (4.5%)	6 (27.3%)	0 (0.0%)	1 (1.1%)
Treatment-related TEAEs leading to death	1 (0.6%)	0 (0.0%)	0 (0.0%)	1 (1.1%)
AESI	15 (9.7%)	4 (18.2%)	3 (7.0%)	8 (9.0%)
DILI	6 (3.9%)	1 (4.5%)	1 (2.3%)	4 (4.5%)
Infusion/injection site reactions	10 (6.5%)	2 (9.1%)	1 (2.3%)	7 (7.9%)
Immuno-mediated TEAEs	23 (14.9%)	4 (18.2%)	5 (11.6%)	14 (15.7%)
Immuno-mediated SAEs	4 (2.6%)	1 (4.5%)	2 (4.7%)	1 (1.1%)

Immuno-mediated AEs are those adverse events consistent with an immune-mediated mechanism of action requiring treatment with systemic corticosteroids or hormone replacement therapy.

Abbreviations: AESI, adverse events of special interest; DILI, drug-induced liver injury; SAE, serious adverse events; TEAEs, treatment-emergent adverse events.

imAEs were reported in 14.9% of patients and were serious in 2.6%, without important differences between subgroups based on the length of induction. imTEAEs by SOC, PT, and maximum grade, overall and by subgroups, are presented in Supplementary Table S3. The most common imTEAEs in the overall safety set were: hypothyroidism, diarrhea, asthenia, and pruritus, all with 3 events in 3 patients (1.9%). The 4 serious imTEAEs consisted of diarrhea, platelet count decreased, encephalitis autoimmune, and pruritus.

Fatal events occurred in 7 (4.5%) patients, 6 of which received ≤3 cycles of induction. One treatment-related fatal event (pneumonia) occurred in 1 patient who received 5-6 cycles of induction: the event was considered as related to carboplatin and etoposide but not related to atezolizumab.

**Figure 1.**
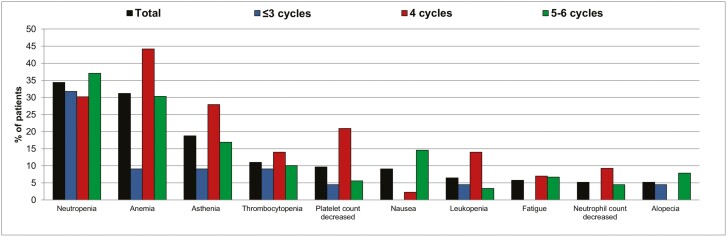
Most common (ie, reported in ≥5% of patients overall) treatment-related (any component) TEAEs by PT.

Treatment-related TEAEs were reported in 76.0% of patients and were grades 3-4 in 47.4%. Figure 1 shows the most common (ie, reported in ≥5% of patients overall) treatment-related (to any component) TEAEs by PT.

AESI was reported in 9.7% of patients overall, and was more common in patients who received ≤3 cycles (18.2%) than in those who received 4 cycles (7.0%) or 5-6 cycles (9.0%), and were related to atezolizumab in 4 (18.2%), 3 (7.0%), and 6 (6.7%) patients, respectively in the 3 subgroups. Infusion/injection site reactions were reported in 6.5% of patients overall and DILI was reported in 3.9% of patients.

TEAEs that caused treatment discontinuation also occurred more frequently in patients who received ≤3 cycles than in the other 2 subgroups. Other subgroup analyses by number of treatment cycles in the induction phase showed that the incidence of TEAEs, treatment-related TEAEs, grade 3-4 TEAEs, grade 3-4 treatment-related TEAEs, and DILI was not related to the number of cycles of induction.

### Efficacy

In the overall ITT population, 65 patients (41.9%; 95% CI, 34.5%-49.8%) were alive at 1 year. The number of patients alive at 1 year was 18 (27.3%; 95% CI, 18.0%-39.0%) in those who received 4 induction cycles and 47 (52.8%; 95% CI, 42.5%-62.8%) in those who received 5-6 cycles. None of the 23 patients that received ≤3 cycles were alive at 1 year.

**Figure 2.**
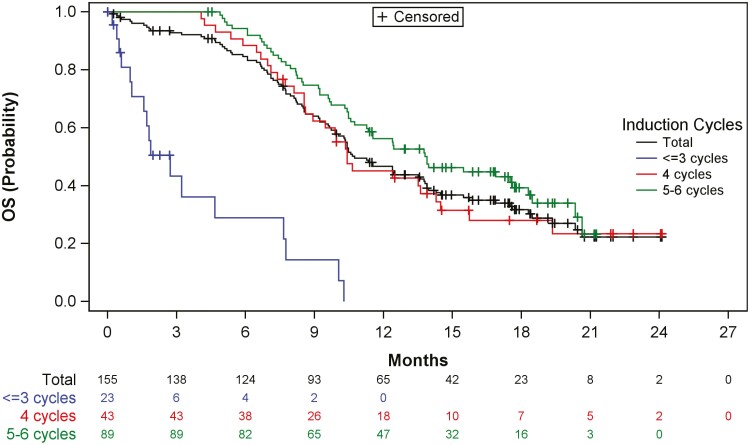
Summary of results of OS overall and by subgroup: Kaplan-Meier estimate (ITT set). Abbreviation: OS, overall survival.

The median OS in the overall ITT population was 10.7 months (95% CI, 9.9-13.7 months). Figure 2 shows the results of OS by subgroup (number of cycles of induction). The median OS was numerically longer in patients who received 5-6 induction cycles (13.8 months, 95% CI, 10.7-18.2 months) than in those who received 4 (10.4 months, 95% CI, 8.6-14.2 months) or ≤3 cycles (2.7 months, 95% CI, 1.0-7.6 months). The median OS in patients who entered the maintenance phase was 13.8 months (95% CI, 11.2-17.6 months). Overall, 101 patients (65.2% of ITT set) died. The proportion of patients who are deceased was higher in those who received ≤3 cycles (17 patients, 73.9%) than in those who received 4 (30 patients, 69.8%) or 5-6 cycles (54 patients, 60.7%). See Supplementary Fig. S2 for OS in patients entering the maintenance phase by tumor response in the induction phase.

**Figure 3.**
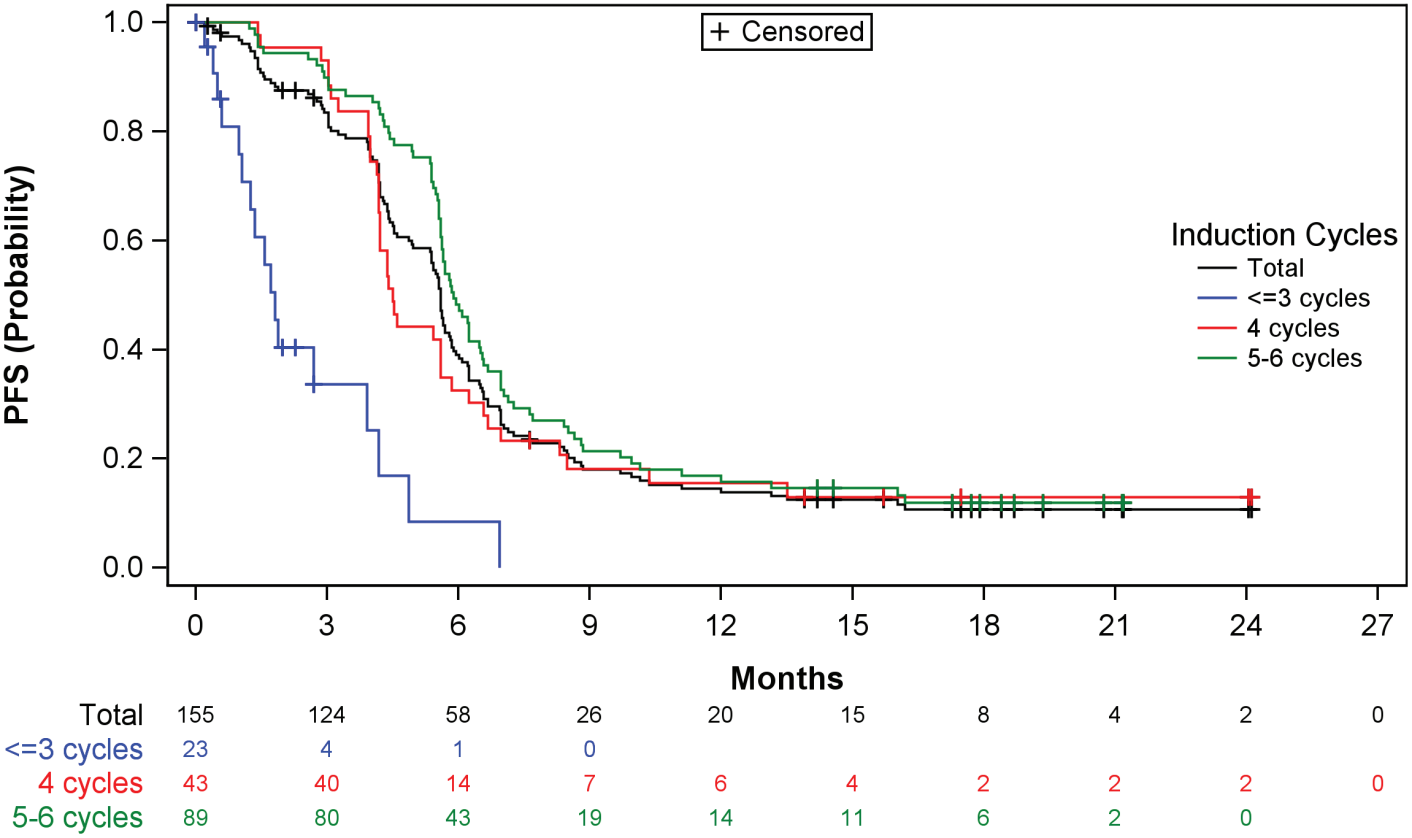
Summary of results of PFS overall and by subgroup: Kaplan-Meier estimate (ITT set). Abbreviation: PFS, progression-free survival.

The median PFS in the overall ITT population was 5.5 months (95% CI, 5.3-5.8 months). Figure 3 shows the results of PFS by subgroup (number of cycles of induction). The median PFS was longer in patients who received 5-6 induction cycles (5.8 months, 95% CI, 5.5-6.5 months) than in those who received 4 (4.5 months, 95% CI, 4.1-5.5 months) or ≤3 cycles (1.8 months, 95% CI, 1.0-3.9 months). The median PFS in patients who entered the maintenance phase was 5.8 months (95% CI, 5.5-6.4 months). Overall, 132 patients (85.2%) had tumor progression. The proportion of patients with progression was higher in those who received 5-6 (78 patients, 87.6%) or 4 cycles (37 patients, 86.0%) than in those who received ≤3 cycles (17 patients, 73.9%).

In the overall ITT population, 111 patients (71.6%; 95% CI, 64.1%-78.1%) had an objective response and 44 (28.4%; 95% CI, 21.9%-35.9%) did not respond to treatment. The proportion of responder patients was higher in those who received 5-6 (75 patients, 84.3%) than in those who received 4 cycles (31 patients, 72.1%) or ≤ 3 cycles (5 patients, 21.7%). The best responses are detailed in Supplementary Table S4.

## Discussion

The IMpower133 study, showing for the first time an improved OS and PFS with the addition of atezolizumab to PE chemotherapy, has set a new standard in the treatment of ES-SCLC. However, the question of whether these results are reproducible in “real world” clinical practice where a less-selected ES-SCLC patient population is treated is still unanswered.

The MAURIS study was designed to test the hypothesis that the IMpower133 regimen has a similar efficacy and safety profile in this setting. Patient enrollment covered a period between the approval of the new indication (EMA approval, September 2019)^21^ and drug availability at the national level (AIFA reimbursement, July 2020).^22^

This pre-planned interim data analysis was conducted to assess the safety of the treatment regimen in the induction phase and preliminary data of efficacy at 1 year after the closure of the enrolment.

In this study, chemotherapy could be continued for up to 6 cycles according to Investigator’s choice based on safety and efficacy evaluation after 4 cycles of induction. Other differences from the IMpower133 trial^9^ were the possibility to include patients with ECOG PS of 2 (6 patients, 3.9%) and untreated brain metastases (13 patients, 8.4%). The baseline characteristics of patients show that, compared to the IMpower133, the study included more patients with liver (56% vs. 37%) and brain metastases (12% vs. 9%), and fewer patients with prior cancer-related surgery (7.1 vs. 16.4%). Moreover, liver metastases at inclusion, a factor known to be associated with increased risk of bone metastases^23^ and mortality in SCLC,^24^ were more common in this study (55.5% of patients) than in IMpower133 (37%). Considering the effects of ECOG PS^25,26^ and brain metastases and associated treatment^27,28^ on survival outcomes in ES-SCLC patents, the entry criteria and the baseline data suggest that participants in the MAURIS study had a worse prognosis than those of the IMpower133.^12^

More than half of patients continued induction following 4 cycles and only 23 patients were able to complete ≤3 cycles of chemotherapy, mainly due to rapid disease progression. The results of safety in the induction phase of the study are in line with the known safety profile of the treatment regimen used in the study. SAEs were reported in 29.9% of patients and were considered to be related to treatment in 18.2%. Although a direct full comparison with safety data of the overall IMpower133 trial cannot be made, we can observe that the % SAEs was similar in both studies (29% for IMpower133).^10^ The lower rates of SAEs, treatment-related SAEs, treatment-related TEAEs, and grades 3-4 treatment-related TEAEs observed in this interim analysis compared to the atezolizumab arm of the IMpower133 trial^9^ are not definitely indicative of better safety profile, however, it can be observed that rates of the most common chemotherapy-induced hematologic toxicities were similar in the 2 studies. In this study, imAEs were reported in 14.9% of patients and were serious in 2.6%. Compared to data limited to the induction phase of the IMpower133 trial,^10^ in which induction was limited to 4 cycles, imAEs, overall and in each category, were reported in lower rates of patients. Moreover, none of the imAEs by PT in this study was reported in more than 2% of patients and only 4 imAEs were serious.

With regard to the analysis of safety data by number of cycles of induction, the incidence of serious TEAEs and serious TEAEs related to the study drug was lower in patients who completed 5-6 cycles of induction than in the other 2 categories, whereas TEAEs related to the study drug, AESI and imAEs were reported in similar rates in patients completing 5-6 or 4 cycles of induction. Notably, 11 out of 19 (57.9%) with brain metastases and 3 out of 6 (50.0%) patients with ECOG PS 2 received 5 or 6 cycles of induction, thus suggesting that prolongation of the induction phase may be feasible in these subgroups. However, caution should be used in the interpretation of data on safety in the subgroup analysis based on the number of treatment cycles in the induction phase, considering that approximately 58% of patients treated with atezolizumab received 5-6 cycles of induction and that only approximately 15% of patients received ≤3 cycles. It can be presumed that in patients who received ≤3 cycles, treatment was prematurely interrupted due to TEAEs or rapid tumor progression and safety in each patient had a key role in the decision to prolong the number of cycles of induction. The results of efficacy observed up to the cutoff date (median follow-up 10.5 months, range 0.2-24.3 months) seem to be in line with those observed in the IMpower133 trial.^9^ The median OS (10.7 months, 95% CI, 9.9-13.7 months) was slightly shorter than that of the IMpower133 (12.3 months), and the survival rate at 1 year was lower (41.9% vs 51.7%), possibly due to the inclusion of patients with a worse prognosis, for example, ECOG PS 2, untreated brain metastases, the proportion of patients with liver metastases. Notably, the median OS in patients that entered the maintenance phase was longer than that of the overall study population. Conversely, the median PFS (5.5 months) was slightly longer than that of the IMpower133 (5.2 months) and ORR (71.6%) was also higher (60.2% in the atezolizumab arm of the IMpower133). Again, the comparability of efficacy results in the 2 studies is limited by a different length of follow-up (10.5 months and 12.3 months, respectively in the MAURIS and IMpower133 study).

The analysis of efficacy by subgroups of the length of induction showed that the median OS and PFS did appear numerically longer in patients who received 5-6 induction cycles than in those who received 4 cycles and was shortest in those who completed ≤3 cycles. A similar behavior was observed in response rate, thus suggesting that, in addition to safety, efficacy in response after the 4 cycles of induction might have played a role in the decision on the length of induction. However, the longer benefit for patients receiving 5-6 cycles of chemotherapy in comparison with those ≤4 is to be considered only hypothesis-generating and it deserves additional investigation due to the potential selection bias for patients receiving more chemotherapy cycles.

## Conclusion

The MAURIS study provides preliminary evidence that the safety and efficacy of the IMpower133 regimen may be replicable in a population of ES-SCLC with more relaxed selection criteria, closer to that of “real life” clinical practice. Complete data will be made available with the final 3-year analysis of the study.

## Supplementary Material

Supplementary material is available at *The Oncologist* online.

oyad342_suppl_Supplementary_Material

## Data Availability

The datasets generated and/or analyzed during the current study are available from the corresponding author on reasonable request. Requests for the data underlying this publication require a detailed, hypothesis-driven statistical analysis plan that is collaboratively developed by the requestor and company subject matter experts. For up-to-date details on Roche’s Global Policy on the Sharing of Clinical Information and how to request access to related clinical study documents, see here: https://www.roche.com/innovation/process/clinical-trials/data-sharing/. Anonymized records for individual patients across more than one data source external to Roche cannot, and should not, be linked due to a potential increase in the risk of patient re-identification.
