# Supplementary material for: Atezolizumab Plus Carboplatin and Etoposide in Patients with Untreated Extensive-Stage Small-Cell Lung Cancer: Interim Results of the MAURIS Phase IIIb Trial
Source: Oncologist. 2024 Feb 20;29(5):e690–8. doi: 10.1093/oncolo/oyad342 (PMC11067795; doi:10.1093/oncolo/oyad342)
Supplement: oyad342_suppl_Supplementary_Material [file oyad342_suppl_supplementary_material.docx]

**Atezolizumab plus Carboplatin and Etoposide in patients with untreated extensive-stage small-cell lung cancer: results of the MAURIS phase IIIb trial**

**Supplementary Material**

## Contents

[Inclusion and exclusion criteria 1](#_Toc147256056)

[Immune-mediated adverse events 7](#_Toc147256057)

[Adverse events of special interest (AESI) 8](#_Toc147256058)

[Adverse events associated with drug-induced liver injury (DILI) 10](#_Toc147256059)

[Table 1. Reason of discontinuation from treatment and of discontinuations from study (data 11](#_Toc147256060)

[are number and percentage of patients) 11](#_Toc147256061)

[Table 2. Serious TEAEs by SOC, PT and maximum grade 12](#_Toc147256062)

[Table 3. Immune-mediated TEAEs by SOC, PT and maximum grade (Overall and by subgroups) 14](#_Toc147256063)

[Table 4 - Best Response according to induction number of cycles 18](#_Toc147256064)

[Figure 1. Patients’ disposition 19](#_Toc147256065)

[Figure 2. Overall Survival in Patients Entering the Maintenance Phase by Tumour Response in Induction 20](#_Toc147256066)

[Phase / ITT 20](#_Toc147256067)

[Ethics approval and consent to participate 21](#_Toc147256068)

# Inclusion and exclusion criteria

## Inclusion criteria

- Signed Informed Consent Form
- Age ≥ 18 years at time of signing Informed Consent Form
- Ability to comply with the study protocol, in the investigator's judgment
- Histologically or cytologically confirmed ES-SCLC per the Veterans Administration Lung Study Group (VALG) staging system
- Measurable disease, as defined by RECIST v1.1. Previously irradiated lesions could only be considered as measurable disease if disease progression had been unequivocally documented at that site since radiation and the previously irradiated lesion was not the only site of disease
- Eastern Cooperative Oncology Group (ECOG) performance status (PS) from 0 to 2
- Life expectancy > 12 weeks
- No prior systemic treatment for ES-SCLC
- Patients who had received prior chemoradiotherapy for limited-stage SCLC had to be been treated with curative intent and experienced a treatment-free interval of at least 6 months since last chemotherapy, radiotherapy, or chemoradiotherapy cycle from diagnosis of ES-SCLC
- Patients where thoracic radiotherapy (consolidation RT) was clinically indicated could be enrolled providing they received RT between the completion of induction phase and the beginning of maintenance phase
- Patients with paraneoplastic syndromes could be enrolled if an autoimmune origin could be excluded
- Adequate hematologic and end organ function, defined by the following laboratory results, obtained within 14 days prior to initiation of study treatment:
  - ANC ≥1.5 × 10^9^/L (1500/μL) without granulocyte colony-stimulating factor support
  - Lymphocyte count *≥* 0.5 × 10^9^/L (500/μL)
  - Platelet count ≥100 × 10^9^/L (100,000/μL) without transfusion
  - Hemoglobin ≥90 g/L (9 g/dL)

Patients could be transfused to meet this criterion

- - AST, ALT, and alkaline phosphatase (ALP) ≤ 2.5 x upper limit of normal (ULN), with the following exceptions:

Patients with documented liver metastases: AST and/or ALT ≤ 5 x ULN

Patients with documented liver or bone metastases: ALP ≤ 5 x ULN.

- - Bilirubin ≤1.25 x ULN with the following exception:

Patients with known Gilbert disease: serum bilirubin level ≤ 3 x ULN

- - Creatinine ≤1.5 x ULN
  - Albumin ≥ 25 g/L (2.5 g/dL)
  - For patients not receiving therapeutic anticoagulation: International Normalised ratio (INR) or activated partial thromboplastin time (aPTT) ≤ 1.5 × ULN
  - For patients receiving therapeutic anticoagulation: stable anticoagulant regimen
- Negative human immunodeficiency virus (HIV) test at screening
- Negative hepatitis B surface antigen (HBsAg) test at screening
- Negative total hepatitis B core antibody (HBcAb) test at screening, or positive total HBcAb test followed by a negative hepatitis B virus (HBV) DNA test at screening

The HBV DNA test was to be performed only for patients who had a positive total HBcAb test.

- Negative hepatitis C virus (HCV) antibody test at screening, or positive HCV antibody test followed by a negative HCV RNA test at screening

The HCV RNA test was performed only for patients who had a positive HCV antibody test.

- For women of childbearing potential: agreement to remain abstinent (refrain from heterosexual intercourse) or use of contraception, as defined below:
  - Women had to remain abstinent or use contraceptive methods with a failure rate of  1% per year during the treatment period and for at least 5 months after the last dose of study treatment. - A woman was considered to be of childbearing potential if she was post-menarcheal, had not reached a postmenopausal state (≥12 continuous months of amenorrhea with no identified cause other than menopause), and was not permanently infertile due to surgery (i.e., removal of ovaries, fallopian tubes, and/or uterus) or another cause as determined by the investigator (e.g., Müllerian agenesis). *Per this definition, a woman with a tubal ligation was considered*

*to be of childbearing potential.* The definition of childbearing potential could be adapted for alignment with local guidelines or regulations.

- - Examples of contraceptive methods with a failure rate of  1% per year included bilateral tubal ligation, male sterilization, hormonal contraceptives that inhibit ovulation, hormone-releasing intrauterine devices, and copper intrauterine devices.
  - The reliability of sexual abstinence should have been evaluated in relation to the duration of the clinical trial and the preferred and usual lifestyle of the patient. Periodic abstinence (e.g., calendar, ovulation, symptothermal, or postovulation methods) and withdrawal were not acceptable methods of contraception.
- For men: agreement to remain abstinent (refrain from heterosexual intercourse) or use a condom, and agreement to refrain from donating sperm, as defined below:
  - With a female partner of childbearing potential or pregnant female partner, men had to remain abstinent or use a condom during treatment with chemotherapy (i.e., carboplatin and etoposide) and for at least 6 months after the final dose of chemotherapy to avoid exposing the embryo. Men had to refrain from donating sperm during this same period.
  - The reliability of sexual abstinence should have been evaluated in relation to the duration of the clinical trial and the preferred and usual lifestyle of the patient. Periodic abstinence (e.g., calendar, ovulation, symptothermal, or postovulation methods) and withdrawal were not acceptable methods of preventing drug exposure.

## Exclusion criteria

- Symptomatic or actively progressing central nervous system (CNS) metastases. Asymptomatic patients with treated or untreated CNS lesions were eligible, provided that all of the following criteria were met:
  - Measurable disease, per RECIST v1.1, had to be present outside the CNS
  - The patient had no history of intracranial hemorrhage or spinal cord hemorrhage
  - The patient had not undergone stereotactic radiotherapy within 7 days prior to initiation of study treatment, whole-brain radiotherapy within 14 days prior to initiation of study treatment, or neurosurgical resection within 28 days prior to initiation of study treatment
  - The patient had no ongoing requirement for corticosteroids as therapy for CNS disease.

Anticonvulsant therapy at a stable dose was permitted.

- - Metastases were limited to the cerebellum or the supratentorial region (i.e., no metastases to the midbrain, pons, medulla, or spinal cord)
  - There was no evidence of interim progression between completion of CNS directed therapy

(if administered) and initiation of study treatment

- - Asymptomatic patients with CNS metastases newly detected at screening were allowed at

Investigator’s discretion with no need to repeat the screening brain scan

- History of leptomeningeal disease
- Uncontrolled tumor-related pain
  - Patients requiring pain medication had to be on a stable regimen at study entry
  - Symptomatic lesions (e.g., bone metastases or metastases causing nerve impingement) amenable to palliative radiotherapy should have been treated prior to enrollment. Patients should have been recovered from the effects of radiation. There was no required minimum recovery period
  - Asymptomatic metastatic lesions that would likely cause functional deficits or intractable pain with further growth (e.g., epidural metastasis that is not currently associated with spinal cord compression) should be considered for loco-regional therapy if appropriate prior to enrolment.
  - Uncontrolled pleural effusion, pericardial effusion, or ascites requiring recurrent drainage procedures (once monthly or more frequently). Patients with indwelling catheters (e.g., PleurX^©^) were allowed regardless of drainage frequency.
- Uncontrolled or symptomatic hypercalcemia (ionized calcium  1.5 mmol/L, calcium  12 mg/dL or corrected calcium *greater than* ULN)
- Active or history of autoimmune disease or immune deficiency, including, but not limited to, myasthenia gravis, myositis, autoimmune hepatitis, systemic lupus erythematosus, rheumatoid arthritis, inflammatory bowel disease, antiphospholipid antibody syndrome, Wegener granulomatosis, Sjögren syndrome, Guillain-Barré syndrome, or multiple sclerosis, with the following exceptions:
  - Patients with a history of autoimmune-related hypothyroidism who were on thyroid-replacement hormone were eligible for the study.
  - Patients with controlled Type 1 diabetes mellitus who were on an insulin regimen were eligible for the study.
  - Patients with eczema, psoriasis, lichen simplex chronicus, or vitiligo with dermatologic manifestations only (e.g., patients with psoriatic arthritis are excluded) were eligible for the study provided all of following conditions were met: i) rash had to cover less than 10% of body surface area; ii) disease was well controlled at baseline and requires only low-potency topical corticosteroids; iii) there was no *n*o occurrence of acute exacerbations of the underlying condition requiring psoralen plus ultraviolet A radiation, methotrexate, retinoids, biologic agents, oral calcineurin inhibitors, or high-potency or oral corticosteroids within the previous 12 months
  - History of idiopathic pulmonary fibrosis, organizing pneumonia (e.g., bronchiolitis obliterans), drug-induced pneumonitis, idiopathic pneumonitis, or evidence of active pneumonitis on screening chest computerized tomography (CT) scan. History of radiation pneumonitis in the radiation field (fibrosis) was permitted
- Active tuberculosis
- Significant cardiovascular disease, (such as New York Heart Association Class II or greater cardiac disease, myocardial infarction, or cerebrovascular accident) within 3 months prior to initiation of study treatment, unstable arrhythmia, or unstable angina
- Major surgical procedure other than for diagnosis within 4 weeks prior to initiation of study treatment, or anticipation of need for a major surgical procedure during the study
- History of malignancy other than SCLC within 5 years prior to screening, with the exception of malignancies with a negligible risk of metastasis or death (e.g., 5-year OS rate  90%), such as adequately treated carcinoma in situ of the cervix, non-melanoma skin carcinoma, localized prostate cancer, ductal carcinoma in situ, or Stage I uterine cancer
- Severe infection within 4 weeks prior to initiation of study treatment, including, but not limited to, hospitalization for complications of infection, bacteremia, or severe pneumonia
- Treatment with therapeutic oral or IV antibiotics within 2 weeks prior to initiation of study treatment. Patients receiving prophylactic antibiotics (e.g., to prevent a urinary tract infection or chronic obstructive pulmonary disease exacerbation) were eligible for the study
- Prior allogeneic stem cell or solid organ transplantation
- Any other diseases, metabolic dysfunction, physical examination finding, or clinical laboratory finding that contraindicated the use of an investigational drug, could affect the interpretation of the results, or could render the patient at high risk from treatment complications
- Treatment with a live, attenuated vaccine within 4 weeks prior to initiation of study treatment, or anticipation of need for such a vaccine during atezolizumab treatment or within 5 months after the final dose of atezolizumab
- Current treatment with anti-viral therapy for HBV
- Treatment with investigational therapy within 28 days prior to initiation of study treatment
- Prior treatment with CD137 agonists or immune checkpoint blockade therapies, including anti−CTLA-4, anti−PD-1, and anti−PD-L1 therapeutic antibodies
- Treatment with systemic immunostimulatory agents (including, but not limited to, interferon and interleukin 2 [IL-2]) within 4 weeks or 5 drug elimination half-lives (whichever is longer) prior to initiation of study treatment
- Treatment with systemic immunosuppressive medication (including, but not limited to, corticosteroids, cyclophosphamide, azathioprine, methotrexate, thalidomide, and anti−*tumor necrosis factor*-α *[TNF-*α*]* agents) within 2 weeks prior to initiation of study treatment, or anticipation of need for systemic immunosuppressive medication during study treatment, with the following exceptions:
  - Patients who received acute, low-dose systemic immunosuppressant medication or a one-time pulse dose of systemic immunosuppressant medication (e.g., 48 hours of corticosteroids for a contrast allergy) are eligible for the study
  - Patients who received mineralocorticoids (e.g., fludrocortisone), corticosteroids for chronic obstructive pulmonary disease (COPD) or asthma, or low-dose corticosteroids for orthostatic hypotension or adrenal insufficiency are eligible for the study.
- History of severe allergic anaphylactic reactions to chimeric or humanized antibodies or fusion proteins
- Known hypersensitivity to Chinese hamster ovary cell products or to any component of the atezolizumab formulation
- Known allergy or hypersensitivity to carboplatin or etoposide
- Pregnancy or breastfeeding, or intention of becoming pregnant during study treatment or within 5 months after the final dose of study treatment. Women of childbearing potential had to have a negative serum pregnancy test result within 14 days prior to initiation of study treatment

# Immune-mediated adverse events

- Pulmonary events: pneumonitis
- Hepatic events: hepatitis
- Gastrointestinal events: diarrhea, colitis
- Endocrine events: hypothyroidism, hyperthyroidism, adrenal insufficiency, diabetes mellitus, hypophysitis
- Ocular events: uveitis, retinal events
- Cardiac events: myocarditis
- Infusion-related reactions and cytokine-release syndrome
- Pancreatic events: pancreatitis
- Dermatologic events: rash, Stevens-Johnson syndrome, toxic epidermal necrolysis
- Neurologic events: myasthenia gravis, Guillain-Barré syndrome, meningoencephalitis
- Renal events: nephritis
- Musculoskeletal events: myositis
- Other immune mediated reactions: hemophagocytic lymphohistiocytosis, macrophage activation syndrome

# Adverse events of special interest (AESI)

AESI were any of the following adverse events:

- Cases of potential drug-induced liver injury that include an elevated ALT or AST in combination with either an elevated bilirubin or clinical jaundice, as defined by Hy's Law
- Suspected transmission of an infectious agent by the study treatment, as defined below

Any organism, virus, or infectious particle (e.g., prion protein transmitting transmissible spongiform encephalopathy), pathogenic or non-pathogenic, is considered an infectious agent. A transmission of an infectious agent may be suspected from clinical symptoms or laboratory findings that indicate an infection in a patient exposed to a medicinal product. This term applies only when a contamination of study treatment is suspected.

- Pneumonitis
- Colitis
- Endocrinopathies: diabetes mellitus, pancreatitis, adrenal insufficiency, hyperthyroidism, and hypophysitis
- Hepatitis, including AST or ALT  10 × ULN
- Systemic lupus erythematosus
- Neurological disorders: Guillain-Barré syndrome, myasthenic syndrome or myasthenia gravis, and meningoencephalitis
- Events suggestive of hypersensitivity, infusion-related reactions, cytokine-release syndrome, influenza-like illness, and systemic inflammatory response syndrome
- Nephritis
- Ocular toxicities (e.g., uveitis, retinitis, optic neuritis)
- Myositis
- Myopathies, including rhabdomyolysis
- Grade ≥ 2 cardiac disorders (e.g., atrial fibrillation, myocarditis, pericarditis)
- Vasculitis
- Autoimmune hemolytic anemia
- Severe cutaneous reactions (e.g., Stevens-Johnson syndrome, dermatitis bullous, toxic epidermal necrolysis)

# Adverse events associated with drug-induced liver injury (DILI)

Adverse event terms (MedDRA PTs) associated with potential DILI were as follows:

- Alanine aminotransferase increased
- Aspartate aminotransferase increased
- Cholestasis
- Drug-induced liver injury
- Hepatic enzyme increased
- Hepatic failure
- Hepatic pain
- Hepatocellular injury
- Hepatomegaly
- Hepatotoxicity
- Hyperbilirubinaemia
- Hypertransaminasaemia
- Jaundice
- Liver disorder
- Liver function test abnormal

# Table 1. Reason of discontinuation from treatment and of discontinuations from study (data

# are number and percentage of patients)

n

%

| Discontinuation from treatment: |  |  |
| --- | --- | --- |
| Adverse event* | 11 | 7.1% |
| Death | 13 | 8.2% |
| Lost to follow-up | 1 | 0.6% |
| Other | 2 | 1.3% |
| Physician decision | 6 | 3.9% |
| Progressive disease | 97 | 63.0% |
| Symptomatic deterioration | 1 | 0.6% |
| Unknown | 1 | 0.6% |
| Withdrawal by subject | 7 | 4.5% |
| Discontinuation from study: |  |  |
| Death | 99 | 64.3% |
| Lost to follow-up | 6 | 3.9% |
| Physician decision | 2 | 1.3% |
| Progressive disease | 1 | 0.6% |
| Withdrawal by subject | 6 | 3.9% |

Percentages refer to the number of treated patients (154)

*The apparent discrepancy with data of Table 2 of the paper (9 patients that discontinued the treatment due to TEAEs) is due to the fact that data of safety reported in Table 2 are limited to the induction phase of the study

# Table 2. Serious TEAEs by SOC, PT and maximum grade

| Primary System Organ Class Preferred Term |  |  | Grade 1-2 |  | Grade 3 | |  | Grade 4 |  | Grade 5 | |
| --- | --- | --- | --- | --- | --- | --- | --- | --- | --- | --- | --- |
|  | Statistic |  |  |  |  | |  |  |  |  | |
| Blood And Lymphatic System Disorders | n (%) E 95% CI |  |  | 1 (0.6%) 1 | 0.0% - 3.6% | | 15 (9.7%) 17 | 5.5% - 16.1% |  |  | |
| Febrile Neutropenia | n (%) E 95% CI |  |  |  | |  | 2 (1.3%) 2 | 0.2% - 4.7% |  |  |  |
| Leukopenia | n (%) E 95% CI |  |  |  | |  | 1 (0.6%) 1 | 0.0% - 3.6% |  |  |  |
| Neutropenia | n (%) E 95% CI |  |  | 1 (0.6%) 1 | | 0.0% - 3.6% | 12 (7.8%) 12 | 4.0% - 13.6% |  |  |  |
| Pancytopenia | n (%) E 95% CI |  |  |  | |  | 1 (0.6%) 1 | 0.0% - 3.6% |  |  |  |
| Thrombocytopenia | n (%) E 95% CI |  |  |  | |  | 1 (0.6%) 1 | 0.0% - 3.6% |  |  |  |
| Cardiac Disorders | n (%) E 95% CI |  |  | 2 (1.3%) 2 | | 0.2% - 4.7% |  |  | 3 (1.9%) 3 | 0.4% - 5.7% |  |
| Bradycardia | n (%) E 95% CI |  |  | 1 (0.6%) 1 | | 0.0% - 3.6% |  |  |  |  |  |
| Cardiac Failure | n (%) E 95% CI |  |  |  | |  |  |  | 2 (1.3%) 2 | 0.2% - 4.7% |  |
| Cardio-Respiratory Arrest | n (%) E 95% CI |  |  |  | |  |  |  | 1 (0.6%) 1 | 0.0% - 3.6% |  |
| Supraventricular Tachycardia | n (%) E 95% CI |  |  | 1 (0.6%) 1 | | 0.0% - 3.6% |  |  |  |  |  |
| Gastrointestinal Disorders | n (%) E 95% CI | 1 (0.6%) 1 | 0.0% - 3.6% | 2 (1.3%) 2 | | 0.2% - 4.7% |  |  | 1 (0.6%) 1 | 0.0% - 3.6% |  |
| Abdominal Pain Upper | n (%) E 95% CI |  |  | 1 (0.6%) 1 | | 0.0% - 3.6% |  |  |  |  |  |
| Constipation | n (%) E 95% CI |  |  | 1 (0.6%) 1 | | 0.0% - 3.6% |  |  |  |  |  |
| Diarrhea | n (%) E 95% CI | 1 (0.6%) 1 | 0.0% - 3.6% |  | |  |  |  | 1 (0.6%) 1 | 0.0% - 3.6% |  |
| General Disorders And Administration Site Conditions | n (%) E 95% CI | 2 (1.3%) 2 | 0.2% - 4.7% |  | |  |  |  |  |  |  |
| Chest Pain | n (%) E 95% CI | 1 (0.6%) 1 | 0.0% - 3.6% |  | |  |  |  |  |  |  |
| Non-Cardiac Chest Pain | n (%) E 95% CI | 1 (0.6%) 1 | 0.0% - 3.6% |  | |  |  |  |  |  |  |
| Infections And Infestations | n (%) E 95% CI | 3 (1.9%) 3 | 0.4% - 5.7% | 3 (1.9%) 3 | | 0.4% - 5.7% |  |  | 1 (0.6%) 1 | 0.0% - 3.6% |  |
| Lung Abscess | n (%) E 95% CI | 1 (0.6%) 1 | 0.0% - 3.6% |  | |  |  |  |  |  |  |
| Pneumonia | n (%) E 95% CI |  |  | 1 (0.6%) 1 | | 0.0% - 3.6% |  |  | 1 (0.6%) 1 | 0.0% - 3.6% |  |
| Soft Tissue Infection | n (%) E 95% CI |  |  | 1 (0.6%) 1 | | 0.0% - 3.6% |  |  |  |  |  |
| Staphylococcal Infection | n (%) E 95% CI | 1 (0.6%) 1 | 0.0% - 3.6% |  | |  |  |  |  |  |  |
| Urinary Tract Infection | n (%) E 95% CI | 1 (0.6%) 1 | 0.0% - 3.6% | 1 (0.6%) 1 | | 0.0% - 3.6% |  |  |  |  |  |
| Investigations | n (%) E 95% CI |  |  |  | |  | 5 (3.2%) 7 | 1.1% - 7.6% |  |  |  |
| Neutrophil Count Decreased | n (%) E 95% CI |  |  |  | |  | 4 (2.6%) 4 | 0.7% - 6.7% |  |  |  |
| Platelet Count Decreased | n (%) E 95% CI |  |  |  | |  | 2 (1.3%) 2 | 0.2% - 4.7% |  |  |  |
| White Blood Cell Count Decreased | n (%) E 95% CI |  |  |  | |  | 1 (0.6%) 1 | 0.0% - 3.6% |  |  |  |
| Metabolism And Nutrition Disorders | n (%) E 95% CI |  |  | 2 (1.3%) 2 | | 0.2% - 4.7% | 1 (0.6%) 1 | 0.0% - 3.6% | 1 (0.6%) 1 | 0.0% - 3.6% |  |
| Diabetic Complication | n (%) E 95% CI |  |  |  | |  |  |  | 1 (0.6%) 1 | 0.0% - 3.6% |  |
| Hyponatraemia | n (%) E 95% CI |  |  | 2 (1.3%) 2 | | 0.2% - 4.7% | 1 (0.6%) 1 | 0.0% - 3.6% |  |  |  |
| Nervous System Disorders | n (%) E 95% CI | 2 (1.3%) 2 | 0.2% - 4.7% | 3 (1.9%) 3 | | 0.4% - 5.7% | 1 (0.6%) 1 | 0.0% - 3.6% |  |  |  |
| Cerebrovascular Accident | n (%) E 95% CI | 1 (0.6%) 1 | 0.0% - 3.6% |  | |  |  |  |  |  |  |
| Cognitive Disorder | n (%) E 95% CI |  |  | 1 (0.6%) 1 | | 0.0% - 3.6% |  |  |  |  |  |
| Encephalitis Autoimmune | n (%) E 95% CI | 1 (0.6%) 1 | 0.0% - 3.6% |  | |  |  |  |  |  |  |
| Paraesthesia | n (%) E 95% CI |  |  | 1 (0.6%) 1 | | 0.0% - 3.6% |  |  |  |  |  |
| Spinal Cord Compression | n (%) E 95% CI |  |  |  | |  | 1 (0.6%) 1 | 0.0% - 3.6% |  |  |  |
| Syncope | n (%) E 95% CI |  |  | 1 (0.6%) 1 | | 0.0% - 3.6% |  |  |  |  |  |
| Renal And Urinary Disorders | n (%) E 95% CI |  |  |  | |  |  |  | 1 (0.6%) 1 | 0.0% - 3.6% |  |
| Renal Failure | n (%) E 95% CI |  |  |  | |  |  |  | 1 (0.6%) 1 | 0.0% - 3.6% |  |
| Respiratory, Thoracic And Mediastinal Disorders | n (%) E 95% CI |  |  | 2 (1.3%) 2 | | 0.2% - 4.7% | 1 (0.6%) 1 | 0.0% - 3.6% | 1 (0.6%) 1 | 0.0% - 3.6% |  |
| Dyspnoa | n (%) E 95% CI |  |  | 1 (0.6%) 1 | | 0.0% - 3.6% | 1 (0.6%) 1 | 0.0% - 3.6% |  |  |  |
| Pneumonitis | n (%) E 95% CI |  |  | 1 (0.6%) 1 | | 0.0% - 3.6% |  |  |  |  |  |
| Pulmonary Oedema | n (%) E 95% CI |  |  |  | |  |  |  | 1 (0.6%) 1 | 0.0% - 3.6% |  |
| Skin And Subcutaneous Tissue Disorders | n (%) E 95% CI |  |  | 1 (0.6%) 1 | | 0.0% - 3.6% |  |  |  |  |  |
| Pruritus | n (%) E 95% CI |  |  | 1 (0.6%) 1 | | 0.0% - 3.6% |  |  |  |  |  |
| Vascular Disorders | n (%) E 95% CI |  |  | 1 (0.6%) 1 | | 0.0% - 3.6% | 1 (0.6%) 1 | 0.0% - 3.6% |  |  |  |
| Arterial Thrombosis | n (%) E 95% CI |  |  |  | |  | 1 (0.6%) 1 | 0.0% - 3.6% |  |  |  |
| Inferior Vena Cava Syndrome | n (%) E 95% CI |  |  | 1 (0.6%) 1 | | 0.0% - 3.6% |  |  |  |  |  |

Notes:

TESAEs are those reported in the Induction Phase of the study.

N = number of patients, % = Observed percentage, E = number of events, CI = Confidence Interval.

Percentages are calculated relative to the total number of patients in the SAF population.

95% CI of the observed percentage calculated using the Clopper-Pearson methodology, or the Poisson distribution for frequencies below 10%.

If patient experienced more than one TESAE with the same PT or primary SOC, the TESAE with the maximum severity was considered in the analysis.

# Table 3. Immune-mediated TEAEs by SOC, PT and maximum grade (Overall and by subgroups)

|  | | | |  | Grade 1-2 | | | Grade 3 | | Grade 4 | | | Grade 5 | |
| --- | --- | --- | --- | --- | --- | --- | --- | --- | --- | --- | --- | --- | --- | --- |
| Primary System Organ Class Preferred Term | | | | Statistic |  | |  |  | |  | |  |  | |
| Overall | Blood And Lymphatic System Disorders |  | n (%) E 95% CI | 1 (0.6%) 1 | 0.0% - 3.6% | |  |  |  |  | | |  |  |
|  | Anemia |  | n (%) E 95% CI | 1 (0.6%) 1 | 0.0% - 3.6% | |  |  |  |  | | |  |  |
|  | Endocrine Disorders |  | n (%) E 95% CI | 4 (2.6%) 4 | 0.7% - 6.7% | |  |  |  |  | | |  |  |
|  | Hyperthyroidism |  | n (%) E 95% CI | 1 (0.6%) 1 | 0.0% - 3.6% | |  |  |  |  | | |  |  |
|  | Hypothyroidism |  | n (%) E 95% CI | 3 (1.9%) 3 | 0.4% - 5.7% | |  |  |  |  | | |  |  |
|  | Gastrointestinal Disorders |  | n (%) E 95% CI | 3 (1.9%) 3 | 0.4% - 5.7% | |  |  |  |  | | |  |  |
|  | Diarrhea |  | n (%) E 95% CI | 3 (1.9%) 3 | 0.4% - 5.7% | |  |  |  |  | | |  |  |
|  | General Disorders And Administration Site Conditions |  | n (%) E 95% CI | 3 (1.9%) 3 | 0.4% - 5.7% | |  |  |  |  | | |  |  |
|  | Asthenia |  | n (%) E 95% CI | 3 (1.9%) 3 | 0.4% - 5.7% | |  |  |  |  | | |  |  |
|  | Hepatobiliary Disorders |  | n (%) E 95% CI | 1 (0.6%) 1 | 0.0% - 3.6% | | 1 (0.6%) 1 | 0.0% - 3.6% |  |  | | |  |  |
|  | Hypertransaminasaemia |  | n (%) E 95% CI | 1 (0.6%) 1 | 0.0% - 3.6% | | 1 (0.6%) 1 | 0.0% - 3.6% |  |  | | |  |  |
|  | Immune System Disorders |  | n (%) E 95% CI | 1 (0.6%) 1 | 0.0% - 3.6% | |  |  |  |  | | |  |  |
|  | Drug Hypersensitivity |  | n (%) E 95% CI | 1 (0.6%) 1 | 0.0% - 3.6% | |  |  |  |  | | |  |  |
|  | Injury, Poisoning And Procedural Complications |  | n (%) E 95% CI | 2 (1.3%) 2 | 0.2% - 4.7% | |  |  |  |  | | |  |  |
|  | Infusion Related Reaction |  | n (%) E 95% CI | 2 (1.3%) 2 | 0.2% - 4.7% | |  |  |  |  | | |  |  |
|  | Investigations |  | n (%) E 95% CI |  |  | |  |  | 1 (0.6%) 1 | 0.0% - 3.6% | | |  |  |
|  | Platelet Count Decreased |  | n (%) E 95% CI |  |  | |  |  | 1 (0.6%) 1 | 0.0% - 3.6% | | |  |  |
|  | Musculoskeletal And Connective Tissue Disorders | | n (%) E 95% CI | 1 (0.6%) 1 | 0.0% - 3.6% | |  |  |  |  | | |  |  |
|  | Back Pain | | n (%) E 95% CI | 1 (0.6%) 1 | 0.0% - 3.6% | |  |  |  |  | | |  |  |
|  | Nervous System Disorders | | n (%) E 95% CI | 2 (1.3%) 2 | 0.2% - 4.7% | |  |  |  |  | | |  |  |
|  | Encephalitis Autoimmune | | n (%) E 95% CI | 1 (0.6%) 1 | 0.0% - 3.6% | |  |  |  |  | | |  |  |
|  | Paraesthesia | | n (%) E 95% CI | 1 (0.6%) 1 | 0.0% - 3.6% | |  |  |  |  | | |  |  |
|  | Reproductive System And Breast Disorders | | n (%) E 95% CI | 1 (0.6%) 1 | 0.0% - 3.6% | |  |  |  |  | | |  |  |
|  | Amenorrhea | | n (%) E 95% CI | 1 (0.6%) 1 | 0.0% - 3.6% | |  |  |  |  | | |  |  |
|  | Respiratory, Thoracic And Mediastinal Disorders | | n (%) E 95% CI | 1 (0.6%) 1 | 0.0% - 3.6% | |  |  |  |  | | |  |  |
|  | Pneumonitis | | n (%) E 95% CI | 1 (0.6%) 1 | 0.0% - 3.6% | |  |  |  |  | | |  |  |
|  | Skin And Subcutaneous Tissue Disorders | | n (%) E 95% CI | 5 (3.2%) 5 | 1.1% - 7.6% | | 1 (0.6%) 1 | 0.0% - 3.6% |  |  | | |  |  |
|  | Dry Skin | | n (%) E 95% CI | 1 (0.6%) 1 | 0.0% - 3.6% | |  |  |  |  | | |  |  |
|  | Pruritus | | n (%) E 95% CI | 2 (1.3%) 2 | 0.2% - 4.7% | | 1 (0.6%) 1 | 0.0% - 3.6% |  |  | | |  |  |


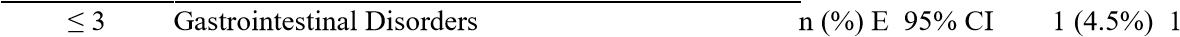
 Rash n (%) E 95% CI 2 (1.3%) 2 0.2% - 4.7%

0.1% - 25.3%

1 (2.3%) 1 0.1% - 13.0%

1 (2.3%) 1 0.1% - 13.0%

| 5-6 | Blood And Lymphatic System Disorders |  | n (%) E 95% CI | 1 (1.1%) 1 | 0.0% - 6.3% |
| --- | --- | --- | --- | --- | --- |
|  | Anaemia |  | n (%) E 95% CI | 1 (1.1%) 1 | 0.0% - 6.3% |
|  | Endocrine Disorders |  | n (%) E 95% CI | 4 (4.5%) 4 | 1.2% - 11.5% |
|  | Hyperthyroidism |  | n (%) E 95% CI | 1 (1.1%) 1 | 0.0% - 6.3% |
|  | Hypothyroidism |  | n (%) E 95% CI | 3 (3.4%) 3 | 0.7% - 9.9% |

| 0.1% - 25.3% |  |  |
| --- | --- | --- |
| 0.1% - 25.3% |  |  |
| 0.1% - 25.3% |  |  |
|  | 1 (4.5%) 1 | 0.1% - 25.3% |
|  | 1 (4.5%) 1 | 0.1% - 25.3% |
| 0.1% - 25.3% |  |  |
| 0.1% - 25.3% |  |  |

Diarrhoea n (%) E 95% CI 1 (4.5%) 1

General Disorders And Administration Site n (%) E 95% CI 1 (4.5%) 1

Conditions

Asthenia n (%) E 95% CI 1 (4.5%) 1

Hepatobiliary Disorders n (%) E 95% CI

Hypertransaminasaemia n (%) E 95% CI

Immune System Disorders n (%) E 95% CI 1 (4.5%) 1

| 4 | General Disorders And Administration Site Conditions | n (%) E 95% CI | 1 (2.3%) 1 | 0.1% - 13.0% |
| --- | --- | --- | --- | --- |
|  | Asthenia | n (%) E 95% CI | 1 (2.3%) 1 | 0.1% - 13.0% |
|  | Investigations | n (%) E 95% CI |  |  |
|  | Platelet Count Decreased | n (%) E 95% CI |  |  |
|  | Nervous System Disorders | n (%) E 95% CI | 1 (2.3%) 1 | 0.1% - 13.0% |
|  | Encephalitis Autoimmune | n (%) E 95% CI | 1 (2.3%) 1 | 0.1% - 13.0% |
|  | Respiratory, Thoracic And Mediastinal Disorders | n (%) E 95% CI | 1 (2.3%) 1 | 0.1% - 13.0% |
|  | Pneumonitis | n (%) E 95% CI | 1 (2.3%) 1 | 0.1% - 13.0% |
|  | Skin And Subcutaneous Tissue Disorders | n (%) E 95% CI | 1 (2.3%) 1 | 0.1% - 13.0% |
|  | Rash | n (%) E 95% CI | 1 (2.3%) 1 | 0.1% - 13.0% |

Drug Hypersensitivity n (%) E 95% CI 1 (4.5%) 1

Notes:

ImTEAEs are those reported in the Induction Phase of the study.

N = number of patients, % = Observed percentage, E = number of events, CI = Confidence Interval.

Percentages are calculated relative to the total number of patients in the SAF population.

|  |  | Grade 1-2 | | Grade 3 | Grade 4 | | Grade 5 |
| --- | --- | --- | --- | --- | --- | --- | --- |
| Primary System Organ Class Preferred Term | Statistic |  |  |  |  |  |  |

| Gastrointestinal Disorders | n (%) E 95% CI | 2 (2.2%) 2 | 0.3% - 8.1% |  |  |
| --- | --- | --- | --- | --- | --- |
| Diarrhoea | n (%) E 95% CI | 2 (2.2%) 2 | 0.3% - 8.1% |  |  |
| General Disorders And Administration Site Conditions | n (%) E 95% CI | 1 (1.1%) 1 | 0.0% - 6.3% |  |  |
| Asthenia | n (%) E 95% CI | 1 (1.1%) 1 | 0.0% - 6.3% |  |  |
| Hepatobiliary Disorders | n (%) E 95% CI | 1 (1.1%) 1 | 0.0% - 6.3% |  |  |
| Hypertransaminasaemia | n (%) E 95% CI | 1 (1.1%) 1 | 0.0% - 6.3% |  |  |
| Injury, Poisoning And Procedural Complications | n (%) E 95% CI | 2 (2.2%) 2 | 0.3% - 8.1% |  |  |
| Infusion Related Reaction | n (%) E 95% CI | 2 (2.2%) 2 | 0.3% - 8.1% |  |  |
| Musculoskeletal And Connective Tissue  Disorders | n (%) E 95% CI | 1 (1.1%) 1 | 0.0% - 6.3% |  |  |
| Back Pain | n (%) E 95% CI | 1 (1.1%) 1 | 0.0% - 6.3% |  |  |
| Nervous System Disorders | n (%) E 95% CI | 1 (1.1%) 1 | 0.0% - 6.3% |  |  |
| Paraesthesia | n (%) E 95% CI | 1 (1.1%) 1 | 0.0% - 6.3% |  |  |
| Reproductive System And Breast Disorders | n (%) E 95% CI | 1 (1.1%) 1 | 0.0% - 6.3% |  |  |
| Amenorrhoea | n (%) E 95% CI | 1 (1.1%) 1 | 0.0% - 6.3% |  |  |
| Skin And Subcutaneous Tissue Disorders | n (%) E 95% CI | 4 (4.5%) 4 | 1.2% - 11.5% | 1 (1.1%) 1 | 0.0% - 6.3% |
| Dry Skin | n (%) E 95% CI | 1 (1.1%) 1 | 0.0% - 6.3% |  |  |
| Pruritus | n (%) E 95% CI | 2 (2.2%) 2 | 0.3% - 8.1% | 1 (1.1%) 1 | 0.0% - 6.3% |
| Rash | n (%) E 95% CI | 1 (1.1%) 1 | 0.0% - 6.3% |  |  |

95% CI of the observed percentage calculated using the Clopper-Pearson methodology, or the Poisson distribution for frequencies below 10%.

If patient experienced more than one ImTEAEs with the same PT or primary SOC, the ImTEAEs with the maximum severity was considered in the analysis.

# Table 4 - Best Response according to induction number of cycles

|  | ≤**3 cycles** | | **4 cycles** | | **5 -6 cycles** | | **Total** | |
| --- | --- | --- | --- | --- | --- | --- | --- | --- |
|  | **n** | ***%*** | **n** | ***%*** | **n** | ***%*** | **n** | ***%*** |
| **Not assessed#** | 10 | *43.5* | - | *0.0* | - | *0.0* | 10 | *6.5* |
| **CR** | - | *0.0* | 1 | *2.3* | 4 | *4.5* | 5 | *3.2* |
| **PR** | 5 | *21.7* | 30 | *69.8* | 71 | *79.8* | 106 | *68.4* |
| **SD** | 6 | *26.1* | 10 | *23.3* | 10 | *11.2* | 26 | *16.8* |
| **PD** | 2 | *8.7* | 2 | *4.7* | 4 | *4.5* | 8 | *5.2* |
| **Total** | 23 | *100.0* | 43 | *100.0* | 89 | *100.0* | 155 | *100.0* |

# Patients did not perform any post-baseline tumor assessment.

Chi-square test: p < 0.0001.

# Figure 1. Patients’ disposition


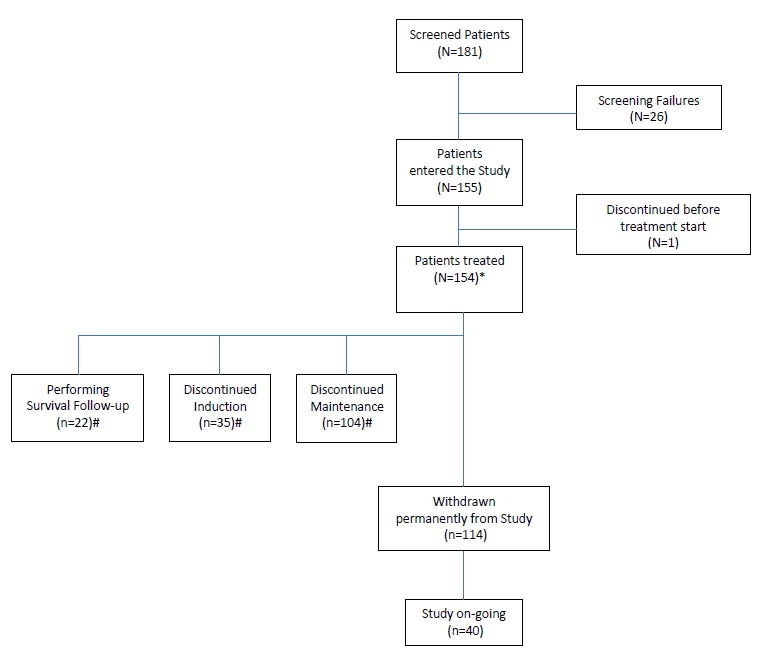


* One patient received treatment with atezolizumab only (no chemotherapy) # A patient may be counted in more than one study phase

|  |  | Median (95% CI) |  | 13.9 (10.4-NE) | 8.6 (7.1 |
| --- | --- | --- | --- | --- | --- |
| Number of patients | Events | n (%) | (59.0%) | (52.2%) | (84.6%) |
|  | Censored | n (%) | (41.0%) | (47.8%) | (15.4%) |

# Figure 2. Overall Survival in Patients Entering the Maintenance Phase by Tumour Response in Induction

Time to events (months)

Statistics

CR+PR

(n=83)

SD

(n=23)

PD

(n=13)

14.4 (11.3

-

19.3)

-

10.3)


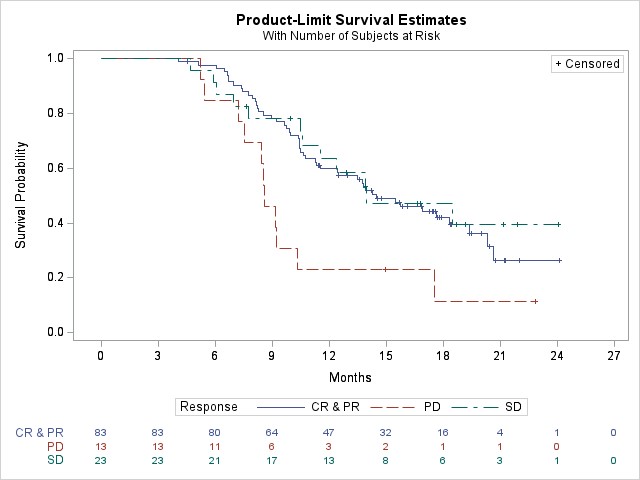


# Phase / ITT

NE = not estimable

# Ethics approval and consent to participate

The study protocol was approved by the reference Ethic Committee of each investigational study site before study start. Patients gave their written informed consent to study participation before the start of any study-related procedure.

Ethics committee that approved the study:

- AOU Vanvitelli –AORN Ospedali dei colli; Comitato Etico Università Vanvitelli, Via Costantinopoli, 104, 80138, Napoli, Campania, ITALY
- Comitato Etico Degli IRCCS Istituto Europeo di Oncologia e Centro Cardiologico Monzino
- Comitato Etico AVEC, VIA ALBERTONI 15, 40138, BOLOGNA, Emilia-Romagna, ITALY
- COMITATO ETICO DELL'UNIVERSITA' CATTOLICA DEL SACRO CUORE – POLICLINICO UNIVERSITARIO A. GEMELLI, Largo Francesco Vito, 10, 00168, Roma, Lazio, ITALY
- Comitato Etico della Fondazione IRCCS Instituto Nazionale dei Tumori, Via G. Venezian, 1, 20133, MILANO, Lombardia, ITALY
- Ce Irccs Onco Basilicata Di Rionero In Vulture, via Padre Pio, 1, Gestione Sperimentale Integrata, 85028, Rionero in Vulture, Basilicata, ITALY
- Comitato Etico Campania Nord, CONTRADA AMORETTA CITTA OSPEDALIERA, 83100, AVELLINO, Campania, ITALY
- Comitato Etico Regionale delle Marche (CERM), Via Conca, 71, 60126, ANCONA, Marche, ITALY
- ARCS – Azienda Regionale di Coordinamento per la Salute, Via Pozzuolo, 330, 33100, Udine, Friuli-Venezia Giulia, ITALY
- Comitato Etico Regione Liguria (Sezione 2), LARGO ROSANNA BENZI 10, 16132, GENOVA, Liguria, ITALY
- Comitato Etico Di Area Vasta Romagna E Irst; Romagnolo per lo Studio e la Cura dei Tumori di Meldola, Via Piero Maroncelli 40, 47014, Meldola, Emilia-Romagna, ITALY
- Comitato Etico Irccs Ospedale San Raffaele, VIA OLGETTINA 60, 20132, MILANO, Lombardia, ITALY
- Comitato Etico IRCCS Pascale
- Comitato Etico Per la Sperimentazione Clinica (CESC) della Provincia di Treviso e Belluno, Via Sant'Ambrogio di Fiera 37, 31100, Treviso, Veneto, ITALY
- Comitato Etico Interaziendale sede presso AOU Maggiore della Carità di Novara, Corso Mazzini 18, 28100, Novara, Piemonte, ITALY
- Comitato Etico Regione Toscana - Area Vasta Nord Ovest, Via Roma 67, c/o Presidio Ospedaliero, 56126, Pisa, Toscana, ITALY
- Comitato Etico A.O. S. Camillo - Forlanini, CIRCONVALLAZIONE GIANICOLENSE 87, 00152, ROMA, Lazio, ITALY
- Comitato Etico Interaziendale della Provincia di Messina, via Consolare Valeria, 98125, Messina, Sicilia, ITALY
- Comitato Etico-Scientifico A.O. Monaldi - Napoli, Via L. Bianchi, 80131, Napoli, Campania, ITALY
- Comitato Etico Area 3 - ASL Lecce
- Comitato Di Bioetica Della ASL Di Sassari, Via Monte Grappa, 82, 07100, Sassari, Sardegna, ITALY
- CESC DELLE PROVINCE DI VERONA E ROVIGO, Piazzale A. Stefani, 1, 37126, Verona, Veneto, ITALY
- COMITATO ETICO PALERMO 2, Viale Strasburgo 233, 90146, Palermo, Sicilia, ITALY
- Comitato Etico della Provincia di Brescia, P.zza Spedali Civili, 1, 25123, Brescia, Lombardia, ITALY
- Università Campus Bio-Medico di Roma, Via Alvaro del Portillo 200, 00128, Roma, Lazio, ITALY

The study was performed in accordance with the Declaration of Helsinki.
